# Maternal undernutrition and the ovine acute phase response to vaccination

**DOI:** 10.1186/1746-6148-4-1

**Published:** 2008-01-15

**Authors:** Peter D Eckersall, Fraser P Lawson, Carol E Kyle, Mary Waterston, Laura Bence, Michael J Stear, Stewart M Rhind

**Affiliations:** 1 Division of Animal Production and Public Health, Institute of Comparative Medicine, Faculty of Veterinary Medicine, University of Glasgow, Bearsden Rd, G61 1QH, UK; 2 Macaulay Institute, Craigiebuckler, Aberdeen, AB15 8QH, UK

## Abstract

**Background:**

The acute phase response is the immediate host response to infection, inflammation and trauma and can be monitored by measuring the acute phase proteins (APP) such as haptoglobin (Hp) or serum amyloid A (SAA). The plane of nutrition during pregnancy is known to affect many mechanisms including the neuroendocrine and neuroimmune systems in neonatal animals but effects on the APP are unknown. To investigate this phenomenon the serum concentration of Hp and SAA was initially determined in non-stimulated lambs from 3 groups (n = 10/group). The dams of the lambs of the respective groups were fed 100% of requirements throughout gestation (High/High; HH); 100% of requirements for the first 65 d of gestation followed by 70% of requirements until 125 d from when they were fed 100% of requirements (High/Low; HL); 65% of liveweight maintenance requirements for the first 65 d gestation followed by 100% of requirements for the remainder of pregnancy (Low/High; LH). The dynamic APP response in the lambs was estimated by measuring the concentration of Hp and SAA following routine vaccination with a multivalent clostridial vaccine with a Pasteurella component, Heptavac P™ following primary and secondary vaccination.

**Results:**

The Hp and SAA concentrations were significantly lower at the time of vaccination (day 8–14) than on the day of birth. Vaccination stimulated the acute phase response in lambs with increases found in both Hp and SAA. Maternal undernutrition led to the SAA response to vaccination being significantly lower in the HL group than in the HH group. The LH group did not differ significantly from either the HH or HL groups. No significant effects of maternal undernutrition were found on the Hp concentrations. A significant reduction was found in all groups in the response of SAA following the second vaccination compared to the response after the primary vaccination but no change occurred in the Hp response.

**Conclusion:**

Decreased SAA concentrations, post-vaccination, in lambs born to ewes on the HL diet shows that maternal undernutrition prior to parturition affects the innate immune system of the offspring. The differences in response of Hp and SAA to primary and secondary vaccinations indicate that the cytokine driven APP response mechanisms vary with individual APP.

## Background

The acute phase proteins (APP) are blood proteins which provide a means to assess the innate immune system's response to disease [1-3]. They are stimulated by the pro-inflammatory cytokines such as interleukin (IL) 1 & 6 and tumour necrosis factor-α (TNFα). The serum concentration of an APP generally increases or decreases by > 25% in response to inflammation, infection and trauma. In ruminants haptoglobin (Hp) and serum amyloid A (SAA) are major APP with 100 or even 1000 fold increases being observed within 24 or 48 h of stimulation [3].

In cattle, Hp is an effective marker for the presence, severity and recovery of animals with mastitis, enteritis, peritonitis, pneumonia endocarditis, endometritis and for monitoring processes such as tail docking and surgical castration [2,3]. Elevations have also been reported in cows with fatty liver syndrome, at parturition, during starvation and following the stress of road transport [4-6]. Similarly SAA has been identified as a marker of inflammation for instance in experimental infections with *Mannheima haemolytica*, with bovine respiratory syncytial virus and in experimental and natural cases of mastitis [7-10]. There has been much less work on the acute phase response in sheep though it is known that Hp and SAA are major APP which are of value as biomarkers of infectious disease and in experimental models of disease in this species [11-13].

Previous studies of the effects of maternal undernutrition in sheep have shown that many systems can be perturbed in the developing embryo, fetus or offspring including gene expression in the oocyte [14], fetal ovarian structure and function [15,16] and hepatic function [17]. There is also evidence from other species that maternal undernutrition can affect the immune system in offspring [18,19].

In order to investigate the dynamics of the acute phase response a procedure was required to allow a consistent stimulation of the pro-inflammatory cytokine network and the acute phase response that was commensurate with the health of the neonates. Following from studies on the inflammatory response to clostridial vaccines in feedlot cattle which reported a post-vaccination rise in serum Hp [20] it was decided to assess the use of the routine husbandry practice of immunising new-born lambs against infection with bacteria such as clostridia and pasteurella (Heptavac P™) as a means to quantify the dynamic acute phase protein response.

## Results

### Maternal diet and the neonatal acute phase protein concentration

The serum concentrations of Hp and SAA were determined, on the day of parturition, in lambs from 3 groups born to ewes subjected to different planes of nutrition during pregnancy. There was no difference (P > 0.05) in the Hp or SAA concentrations between lambs born to dams of the respective groups which were fed either 100% of requirements (maintenance) throughout gestation (High/High; HH), a maintenance ration and then restricted ration (High/Low; HL) or a restricted ration and then a maintenance diet during pregnancy (Low/High, LH).

However, the mean concentrations of the SAA were higher, in all groups, in the samples taken on the day of parturition than in samples taken immediately prior to the primary vaccination, 8–14 days after parturition (Table 1). Similarly the serum Hp concentration declined, reaching the limit of detection or lower in most samples by the time of the first vaccination (Table 2).

**Table 1:** Median and range of SAA concentrations in serum of lambs on day of parturition, median and range SAA concentrations 8–14 days later, immediately pre-vaccination.

	Day	High/High n = 10	High/Low Diet n = 10	Low/High Diet n = 10
Serum amyloid A mg/L (median; range)	0 (parturition)	24;11–45^a^	21;1–101^a^	29;6–126^a^
Serum amyloid A mg/L (mean ± SD)	8–14 (pre-vacc)	1.0;<0.2–33^b**^	0.8;<0.2–33^b**^	1.4;<0.2–40^b*^

^a,b ^differing letters indicate a significant difference between columns at P < 0.01* or P < 0.001**

**Table 2:** Median and range of haptoglobin in serum of lambs on day of parturition and number of lambs per group with detectable Hp immediately pre-vaccination.

	Day	High/High n = 10	High/Low Diet n = 10	Low/High Diet n = 10
Haptoglobin g/L (median: range)	0 (parturition)	0.02;0.01–0.04	0.01;<0.01–0.05	0.02:<0.01–0.09
Haptoglobin	8–14 (pre-vacc)			
Number of samples with detectable Hp		0	1	1
Number of samples at or below detection limit (<0.01 g/L)		10	9	9

### Maternal nutrition and the acute phase protein response to vaccination

**Figure 1:**
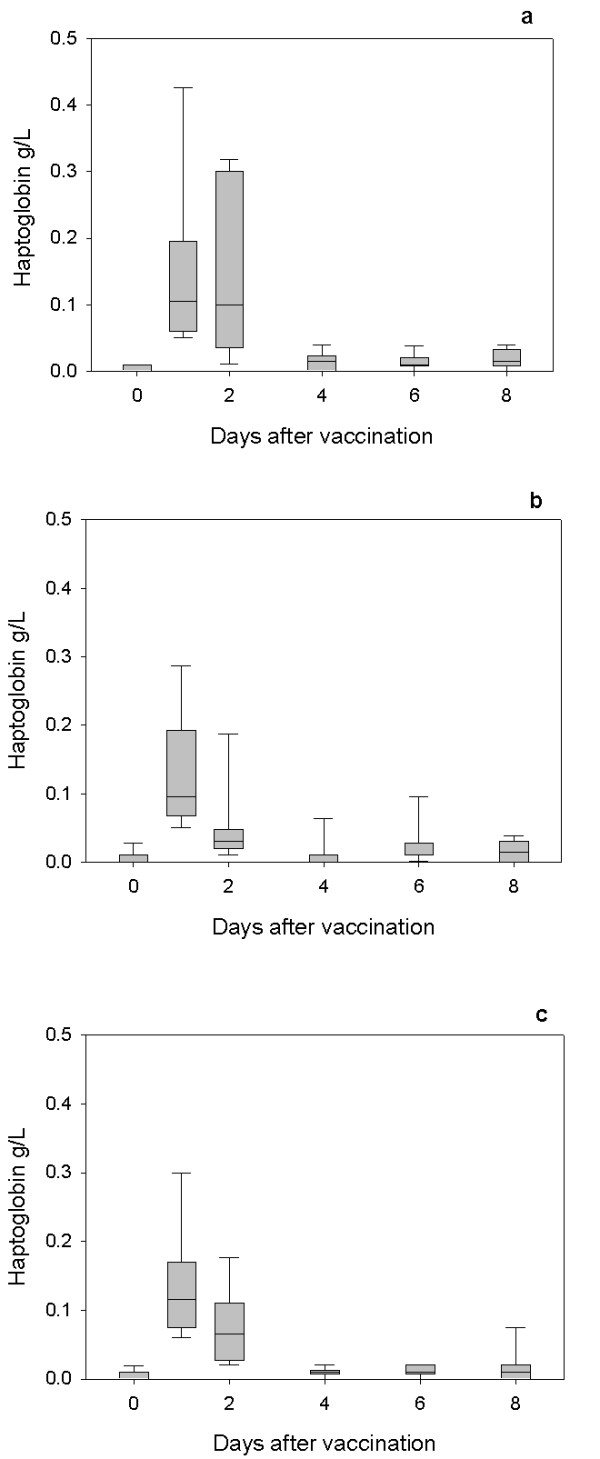
**Hp response to the first vaccination with Heptavac**. The concentrations of haptoglobin in lambs (n = 10) following the first vaccination with Heptavac. The maternal diets of the lambs were High/High (a), High/Low (b) and Low/High (c). The plots show the median as the line in the box, the 25th and 75th percentile are represented by the box with the whiskers representing the 10th and 90th percentiles.

**Figure 2:**
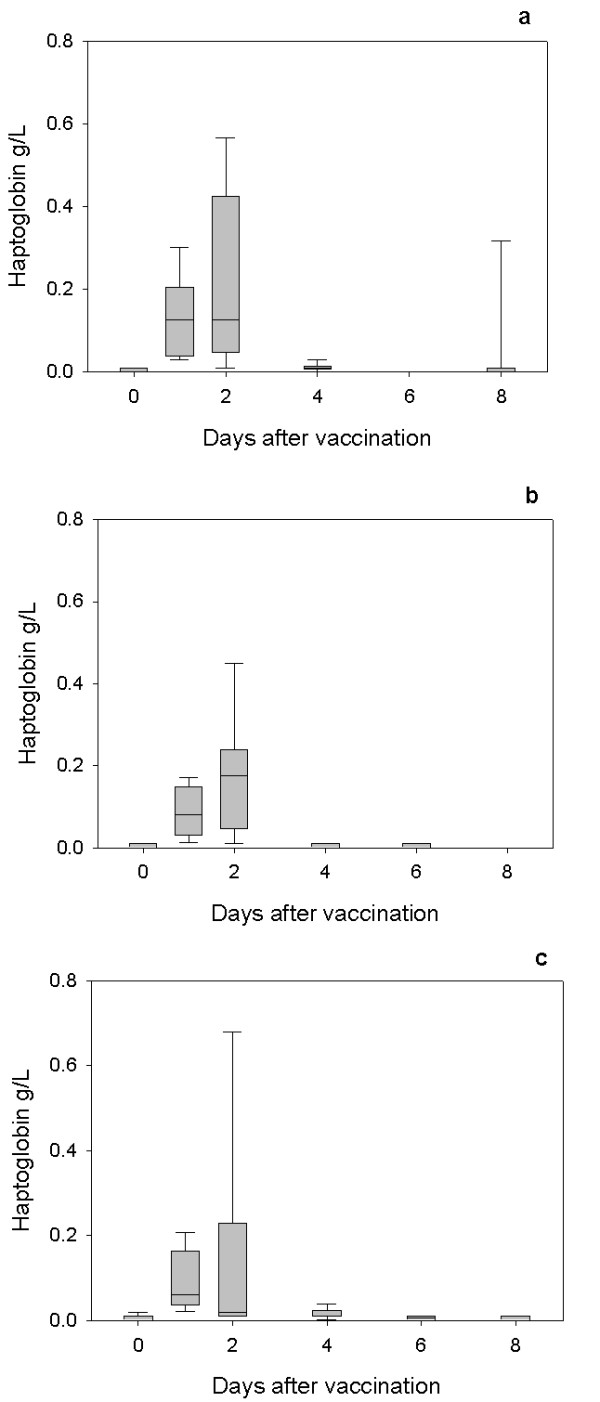
**Hp response to the second vaccination with Heptavac**. The concentrations of haptoglobin in lambs (n = 10) following the second vaccination with Heptavac. The maternal diets of the lambs were High/High (a), High/Low (b) and Low/High (c). The plots show the median as the line in the box, the 25th and 75th percentile are represented by the box with the whiskers representing the 10th and 90th percentiles.

**Figure 3:**
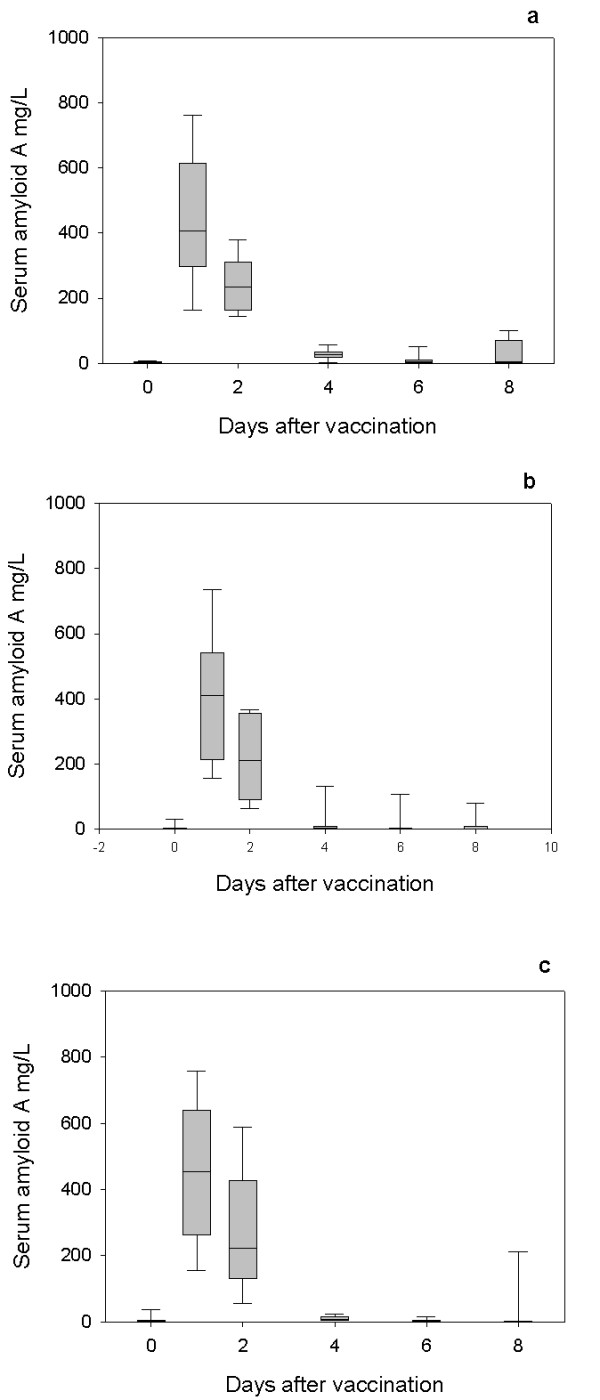
**SAA response to primary vaccination with Heptavac**. The concentrations of serum amyloid A in lambs (n = 10) following the first vaccination with Heptavac. The maternal diets of the lambs were High/High (a), High/Low (b) and Low/High (c). The plots show the median as the line in the box, the 25th and 75th percentile are represented by the box with the whiskers representing the 10th and 90th percentiles.

**Figure 4:**
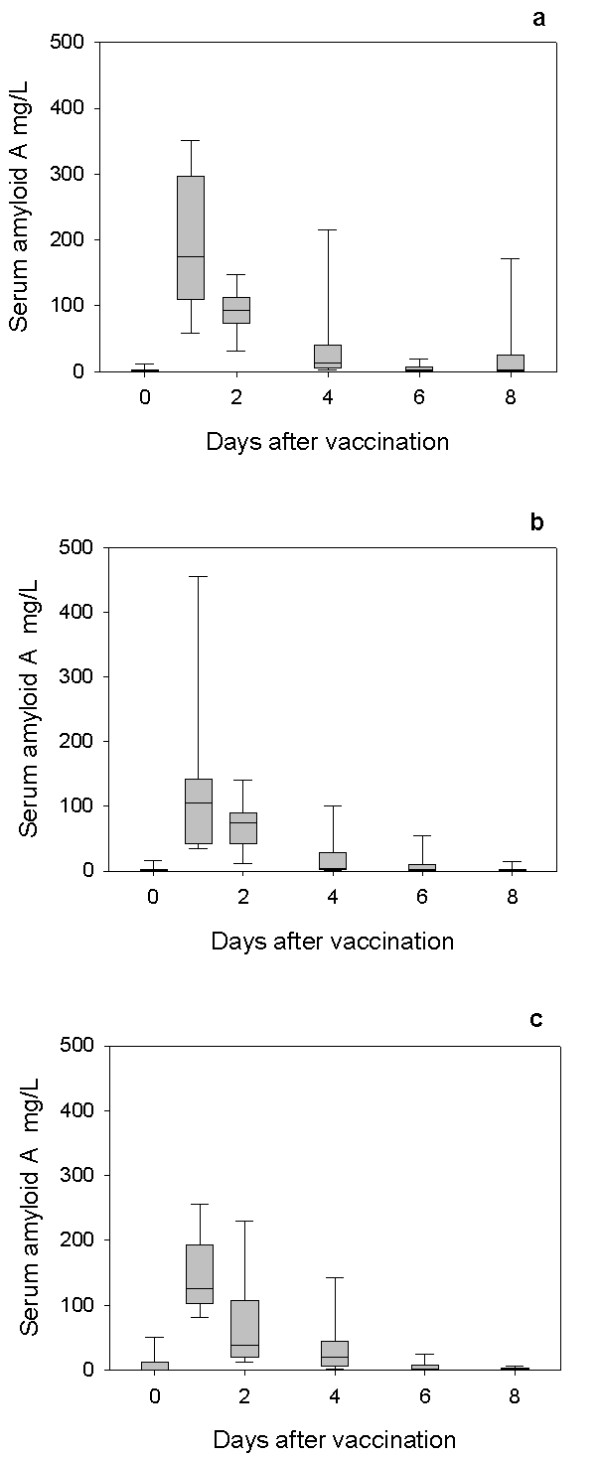
**SAA response to secondary vaccination with Heptavac**. The concentrations of serum amyloid A in lambs (n = 10) following the second vaccination with Heptavac. The maternal diets of the lambs were High/High (a), High/Low (b) and Low/High (c). The plots show the median as the line in the box, the 25th and 75th percentile are represented by the box with the whiskers representing the 10th and 90th percentiles.

All lambs exhibited an acute phase response as demonstrated by increases in serum Hp following first (Figure 1) and second vaccinations (Figure 2) and SAA following the first (Figure 3) and second vaccinations (Figure 4) with Heptavac 7.

For both Hp and SAA, a mixed model analysis using the option of residual maximum likelihood revealed a significant (P < 0.001) effect of day (following vaccination) for both APP. Following the first vaccination, the serum Hp concentration in all groups reached a peak at 24 hours after vaccination (day 1) with median (range) concentrations of 0.11 (0.05–0.43) g/L, 0.10 (0.05–0.29) g/L and 0.12 (0.06–0.31) g/L for the HH, HL and LH nutrition groups respectively (Figures 1a, 1b and 1c). Following the second vaccination, the serum Hp concentration in all groups reached a peak 24 or 48 hours after vaccination with median (range) concentrations of 0.13 (0.02–0.57) g/L, 0.175 (0.02–0.47) g/L and 0.06 (0.02–0.21) g/L for the HH (48 h), HL (48 h) and LH (24 h) nutrition groups respectively (Figures 2a, 2b and 2c).

Following the first vaccination, the SAA concentration in all groups reached a peak 24 hours after vaccination (day 1) with median (range) concentrations of 407 (156–770) mg/L, 411 (153–755) mg/L and 455 (147–765) mg/L for the HH, HL and LH maternal nutrition groups respectively (Figures 3a, 3b and 3c). After the second vaccination, the SAA concentration in all groups also reached a peak 24 hours after vaccination with median (range) concentrations of 175 (54–357) mg/L, 105 (44–481) mg/L and 125 (80–260) mg/L for the HH, HL and LH nutrition groups respectively (Figures 4a, 4b and 4c).

**Figure 5:**
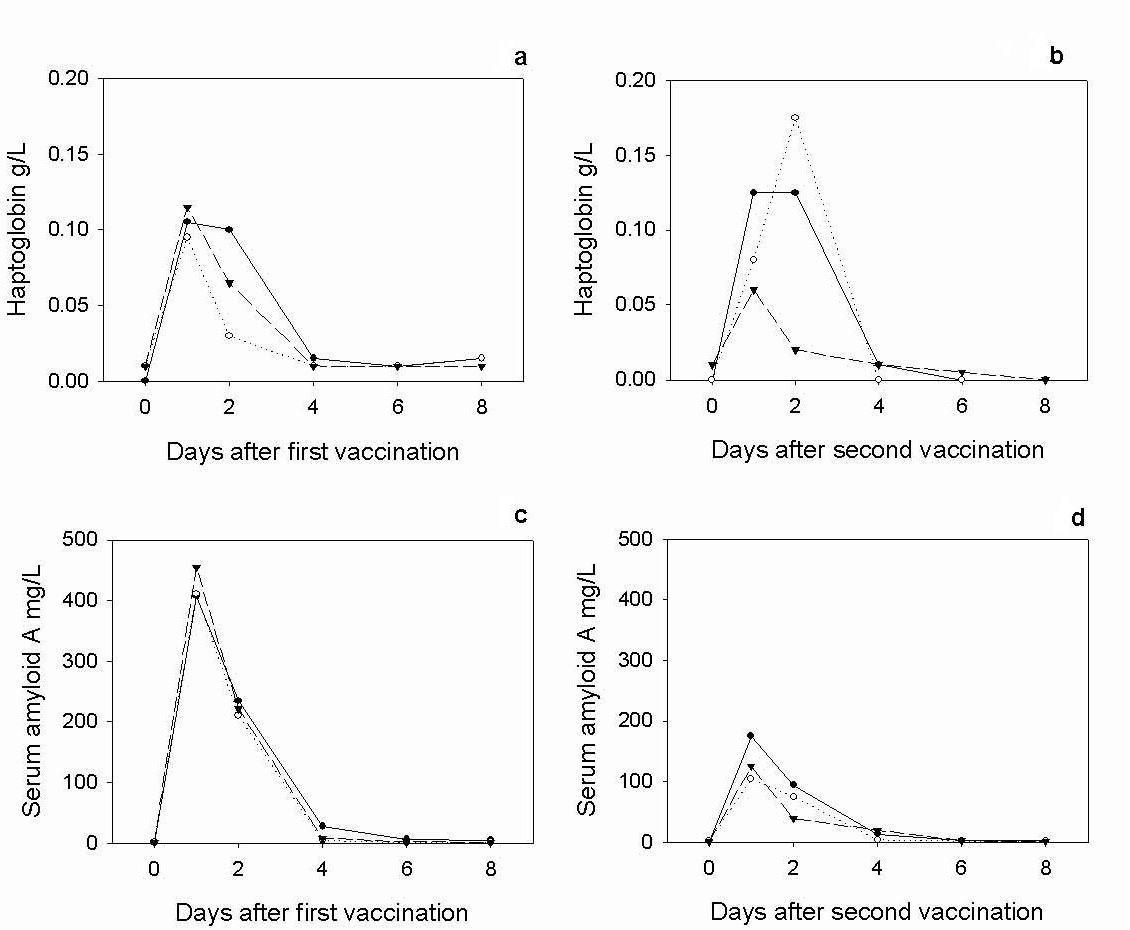
**The median concentrations of Hp and SAA in response to the first and second vaccinations with Heptavac**. The median concentrations of haptoglobin (a and b) and serum amyloid A (c and d) in lambs (n = 10) following the first (a and c) and second (b and d) vaccinations with Heptavac. The maternal diets of the lambs were High/High (closed circle), High/Low (open circle) and Low/High (triangle).

For comparison between the groups and also between responses to vaccinations the medians are plotted on figure 5 showing the Hp response to first (Fig 5a) and second vaccination (Fig 5b) and the SAA response to first (Fig 5c) and second vaccinations (Fig 5d).

The mixed model analysis showed that maternal diet had a significant effect on SAA (P = 0.03) but not Hp (P = 0.13) concentrations. The SAA concentrations were highest in the group with HH maternal diet followed by the LH and HL groups. The SAA in the HH group was significantly greater than in the HL group (P < 0.01) but was not significantly different from that of the LH group and the LH group was not significantly different from the HL group (P > 0.05).

Comparing the primary to the secondary vaccinations using the mixed model analysis showed that the difference in response to each vaccination (P = 0.65) was not significant for Hp whereas there was a significant reduction in SAA concentration following the second vaccinations compared to the primary vaccination (P = 0.01).

The two-way interactions between diet + day (P = 0.524) and diet + vaccine (P = 0.942) and the three way interaction diet + day + vaccine (P = 0.917) were not significant for Hp. However there was a significant day + vaccine interaction (P < 0.001) indicating that the kinetics of the Hp response varied between the first and second vaccinations. For SAA, the two-way interaction between diet + day (P = 0.111), diet + vaccine (P = 0.802) and the three way interaction diet + day + vaccine (P = 0.549) were not significant. There was a highly significant day + vaccine interaction (P < 0.001) indicating that the kinetics of SAA response varied between the first and second vaccinations.

**Figure 6:**
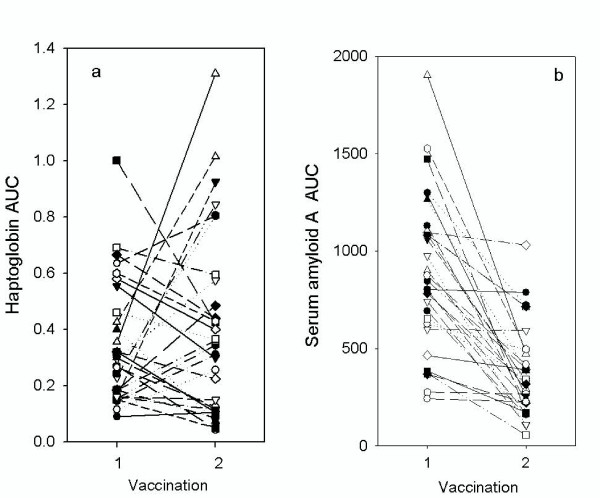
**Area under the curve of APP responses to primary and secondary vaccinations in individual lambs**. The area under the curve calculated for haptoglobin (a) and serum amyloid A (b) for lambs following the first (1) and second (2) vaccination with Heptavac. Each symbol and line represents samples taken from the individual lambs at each vaccination.

Combining all results for the three groups, there was no significant difference in the area under the curve (AUC) of the Hp response following the second (median 0.35 day.g/L range 0.05–1.3 day.g/L) compared to the first (median 0.28 day.g/L; range 0.09–1.0 day.g/L) vaccination. There was no consistent pattern of responses with some lambs having higher (13/30) and some lower (17/30) AUC in the second compared to the primary vaccination (Figure 6a). In contrast, the AUC for the SAA response for the lambs was significantly lower (P < 0.01) after the second vaccination (median 321 day.g/L; range 55–1030 day.mg/L) than after the first vaccination (median 824 day.g/L; range 242–1902 day.mg/L) with the AUC for SAA decreasing in most, but not all, lambs (Figure 6b).

## Discussion

The results of this study show that maternal undernutrition did not cause differences in the APP concentration in lambs at parturition, but that elevated levels of both Hp and SAA were present in all groups with the concentrations falling within 8–14 days. However maternal undernutrition in the HL group, reduced the response of SAA, but not Hp, to vaccination with Heptavac 7™. Apparent differences between groups for Hp concentrations (Fig 5) were not significant due to large variances as shown by the error bars in Figures 1 and 2. Therefore, whereas the restricted diet in the period leading up to parturition caused a reduction in the SAA response, undernutrition in early pregnancy as in the LH group in was not sufficient to affect this part of the innate immune system. This is the first report of the effect of maternal undernutrition on the ability of the acute phase response to contribute to the innate immune defence system but can be viewed as a further example of the potentially detrimental effect that maternal undernutrition has on offspring.

Recent research has demonstrated that maternal undernutrition has multiple and complex effects on the homeostatic and pathophysiological mechanisms in neonates and older offspring [16-18]. A number of these mechanisms could contribute to the effect on the SAA response and also on the difference in SAA response between the first and second vaccinations. For example, maternal undernutrition leads to a reduction in liver size and a reduction in the expression of hepatocyte genes coding for proteins such as growth hormone receptor, prolactin receptor and suppressor of cytokine signalling-3 [17]. As the primary site of synthesis of APP is the liver, following stimulation by pro-inflammatory cytokines, a reduction in liver volume or reduced expression of cytokine receptors could be expected to limit the production of the APP.

Effects of maternal undernutrition have also been observed on the hypothalamo-pituitary-adrenal axis [18]. This has implications for the neuroendocrine-immune axis [21] and this could also influence the responses of the APP to stimulants of the inflammatory and immune response such as the vaccine used here. There are reports of the influence of maternal undernutrition on the cortisol concentrations in offspring; for instance in rats with maternal undernutrition reduced production of cortisol in response to stress in offspring has been reported [18]. Cortisol can have divergent effects on the acute phase protein response, acting synergistically with cytokines to activate the response or reducing the response by acting as an anti-inflammatory agent on macrophage and monocyte production of cytokines [22]. Maternal undernutrition has also been shown in rats, to lead to a lower TNF response to endotoxin [19] which would reduce production of APP, as seen here for SAA. Of interest is the observation, based on unpublished data, from the review by Lesage et al [18] of a significant increase in C-reactive protein, the primary APP in humans, in adults born to undernourished mothers [18]. We found no such significant differences in the pre-vaccination levels of SAA or Hp. However, the relatively small group size (n = 10) and high individual variation in the present study may have contributed to a lack of an observable effect of maternal undernutrition on the non-stimulated APP concentration.

In addition to the consideration of the effects of maternal undernutrition, the study provides further insight into the acute phase response in lambs. The fall in the concentration of Hp and SAA between the day of birth and the day of the primary vaccination (8–14 days after birth) has not been observed previously in this species. However raised levels of acute phase proteins in neonatal animals have been reported in calves and in piglets [23-25] and may be due to the trauma of parturition or colonisation by environmental microbes.

Vaccination with Heptavac 7 caused an acute phase response in all animals. Stimulation of the APP response after vaccination has been reported previously [20] when Hp was the only biochemical or immunological biomarker to show significant change after vaccination of calves with either antigen from multiple clostridial species or with *Clostridium perfingens *types C and D toxoid. Here the study has been extended to quantification of the APP response to vaccination by measuring the reaction of both Hp and SAA and also by comparison of the responses in primary and secondary vaccinations. Monitoring the post-vaccination APP response may be a valuable investigatory tool, both for the examination of the role of the acute phase response in the innate immune system and for the investigation of early vaccine stimulation of the pro-inflammatory cytokines in relation to the efficacy of the vaccination process. Presumably, the immediate post vaccination acute phase response is stimulated by the pro-inflammatory cytokines which are known to be involved in the links between the innate and the acquired immune systems. Indeed research into vaccine adjuvants includes stimulation of the NFκ-B pathway which is also the target of acute phase inducing cytokines such as TNFα [26-28].

The observation of a reduction in the dynamic response of SAA in the second vaccination, relative to that of the response to the primary vaccination, while the haptoglobin response was unchanged relative to the first vaccination, suggests that the control and mechanism of responses for these two proteins are different and could be explained if they are under the control of differing cytokine cocktails [29]. The APP response is an integral part of the innate immune system and would be expected to have responded identically to both primary and secondary vaccinations, though little is known if this is true for stimulators given at 30 day intervals as here. For comparison further studies should include assessment of the response of lambs to a stimulator of sterile inflammation in the absence of vaccine antigens over a similar timeframe. Nevertheless differences were found in the APP response between the two inoculations which may relate to differences in the immunological status of the lambs at each vaccination. The lambs were immunologically naïve at the primary vaccination but not at the time of the secondary vaccination. The reduction in the dynamic acute phase response of SAA to the second vaccination could be explained by the fact that the lambs were partially immune on the latter occasion. It is known that, in humans, SAA is a type I APP, stimulated by IL-1-like cytokines, while Hp is a Type II APP being stimulated by IL-6-like cytokines [29]. If similar mechanisms exist in sheep the primary vaccination could have stimulated both IL-1-like and IL-6-like cytokines while the secondary vaccination had a reduced effect on the IL-1-like cytokines and therefore a reduction the SAA response. Elucidation of the mechanism of differential stimulation of the response of Hp and SAA to primary and secondary vaccination will lead to greater understanding of the role of these APP, and their control by cytokines, in the host defences against infection.

It was not possible in this study to quantify the immune reaction but further investigation of the correlation between specific antibody or cell mediated immunity responses to the Heptavac vaccine and the immediate post-vaccination dynamic acute phase response of Hp or SAA is warranted. If the APP response was found to be correlated with subsequent immunity, monitoring the post vaccination acute phase reaction could have an important role in assessment of vaccine efficacy.

## Conclusion

Maternal undernutrition did not affect the resting concentrations of Hp or SAA in neonatal lambs but these APP were moderately elevated in serum taken on the day of parturition compared to serum taken prior to vaccination at 8–14 days after birth demonstrating that at birth lambs have an elevation of APP concentration. Both first and second vaccination of the lambs with Heptavac vaccine caused an acute phase reaction with increases in the serum concentration of both Hp and SAA. The post vaccination response of SAA but not Hp was affected by maternal undernutrition with the SAA response of the group with dams on the HL diet being significantly lower that the HH group. There was no difference in the response of Hp between vaccinations but there was a significant reduction in the response of SAA in the second vaccination. These results give further weight to findings that maternal undernutrition can have detrimental consequences for offspring, in this case causing a reduction in an important mechanism of the innate immune system. The differences found between the Hp and SAA responses support the conclusion that the APP respond individually to stimulation and relative changes in these APP could be used to identify the relevant cytokine mix at stages of infection. Monitoring the immediate post vaccination acute phase response could also give a valuable and early insight into the efficacy of vaccines.

## Methods

Animal experiments were conducted under the authority of the UK Animals (Scientific Procedures) Act 1986 after Home Office and local ethical committee approval.

### Experimental Design

The ewes used in the study were bought from commercial sources in Scotland such that prior history of vaccination was unknown. Thereafter they were treated with the antihelmintic (Dectomac™ Pfizer Animal Health) but had no vaccination in the 12 months before the lambing in this study.

Ewes were fed variable amounts of a pelleted ration (Green Keil; Harbro Ltd, Turriff, UK; 12.5 MJ metabolisable energy/kg dry matter; 86% dry matter) together with a fixed amount of hay (8.4 MJ/kg dry matter; 85% dry matter), to provide roughage. Ewes of one group (n = 10) were fed 100% of requirements throughout gestation (High/High;H/H). Ewes of the second group (n = 10) were fed 600 g Green Keil and 250 g hay (freshweight) to provide 100% of requirements for the first 65 d of gestation, followed by 70% of requirements until 125 d; amounts of pelleted feed were increased incrementally, at 10 d intervals from day 70, from 380 g/head/d to 640 g/head/d for single-bearing ewes and from 400 to 750 g/head/d for twin-bearing ewes while the hay ration remained constant. After day 125, they were fed 100% of requirements according to stage of gestation (single-bearing: 970–1050 g pellets + hay; twin-bearing: 1130–1230 g pellets + hay), to ensure adequate milk production (High/Low; HL). The ewes of the final group (n = 10) were fed 65% of liveweight maintenance requirements (330 g Green Keil + 250 g hay on a freshweight basis) for the first 65 d gestation followed by 100% of requirements (600 g Green Keil + 250 g hay on a freshweight basis, with the pelleted ration increasing incrementally, every 10 days, according to requirements [30] from 620 g/head/d at day 70 to 1050 g/head/d for single-bearing ewes and from 640 to 1230 g/head/d for twin-bearing ewes), during the remainder of pregnancy (Low/High; LH).

The dynamic acute phase response in the lambs was estimated by measuring the concentration of the APP, following routine vaccination with Heptavac 7™ (Intervet, Milton Keynes, UK), between days 8–14 and at re-vaccination between days 43–49 after birth. The primary immunisation timing was earlier than is normal as it would usually be given at 3 weeks of age but was a requirement of the experimental model in providing protection from infection. Concentrations of APP were determined in serum collected on the day of birth and before each vaccination (day 0) and at 1, 2, 3, 4, 6 and 8 days post-vaccination.

### Acute phase protein measurement

Haptoglobin was measured by the haemoglobin-binding method described by Eckersall et al [31] and SAA was measured by commercial ELISA kit (Tridelta Development, PLC, Dublin, Ireland) according to the manufacturer's instructions.

### Statistical analysis

The significance of differences in mean SAA concentrations between samples taken at parturition and immediately prior to the primary vaccination were determined by the Mann-Whitney test with differences being deemed significant at p < 0.05. The area under the curve (AUC) of the post-vaccination acute phase protein response of haptoglobin and SAA was calculated by the trapezoid rule (SigmaPlot, SPSS Science Software Gmb, Erkrath, Germany) and differences in AUC between the responses to first and second vaccinations were also calculated by the Mann-Whitney test.

Before statistical analysis of the serial samples collected post vaccination, Hp and SAA concentrations were log transformed to meet the requirement for constant variance. As the data conformed to a repeated measures model structure a general linear mixed model (Proc Mixed, SAS Institute, Cary, North Carolina, USA) was used to assess the significance of maternal diet, vaccine (primary or secondary), day (relative to day of vaccination) and associated interactions on the serum concentrations of the acute phase proteins. A residual maximum likelihood analysis was the option used and a separate model was used for Hp and SAA. The equation in matrix notation was:

Y = Xβ + Zu + e

Y is the vector of log.transformed observations on Hp or SAA. X is the incidence matrix of fixed independent variables; diet, vaccine, day and their interactions. β is the vector of experimental errors, Z is the design matrix of random effects; lamb nested within vaccine group and u is the vector of random effects, e is the vector of experimental errors. In these analyses, u was assumed to be distributed multivariate normal with mean vector 0 and covariance matrix G, while e was distributed multivariate normal with mean vector 0 and covariance matrix R. The covariance R was defined to be autoregressive order 1.

## Authors' contributions

PDE stimulated the collaboration and analysed the data. FPL, MW & LB performed the analysis of the acute phase proteins. SMR designed the study of which this work was a component and CEK managed the conduct of the study and the collection of samples. MJS performed the statistical analyses. All authors have read the paper and agree to its publication.
